# Awakening Efforts in Hospital Finance

**Published:** 1919-11-15

**Authors:** 


### The Hospital

The Workers' Newspaper of Administrative Medicine and Institutional Life, Administration, National Insurance and Health.

No. 1745, Vol. LXVII. SATURDAY, NOVEMBER 15, 1919. PRICE TWOPENCE

### AWAKENING EFFORTS IN HOSPITAL FINANCE.

As we foreshadowed last week, circumstances demonstrate that the clearance of the financial atmosphere, so far as those who support hospitals are concerned, by the Finance Debate in the House of Commons, some ten days ago, has improved the outlook for all well-managed hospitals dependent largely on voluntary contributions. The effect has been to encourage well-managed hospitals to organise and evolve new schemes to attract more funds and workers. They recognise that they must strengthen the financial departments of their hospitals. An overhaul of the existing arrangements can lessen, and has lessened, the shortage in income which was feared, so that this promises to be materially reduced in volume, probably before the year is out, and certainly by the first quarter of 1920. Glasgow has appointed a special committee to take in hand and create adequate machinery, whereby the £150,000, required in cash, and the Lister Memorial may both be completed, by energy and zeal, to the glory of Glasgow citizens, the latter by January 1 next, and the former by January 31, 1920. We have further to report the splendid effort made by Mr. Cronshaw and the supporters of the Radcliffe Infirmary at Oxford, whereby this ancient and well-known hospital will extend the area and usefulness of its work, and bring the whole of the institution well up to date. Indeed, Mr. Cronshaw's scheme may set an example to the whole country. The fact that it should have been rapidly brought into being with a contemplated outlay of £60,000, is a striking demonstration of the hold the voluntary hospitals have on the people, throughout the country. Evidence is accumulating which justifies the confidence we have expressed in the plan of reconstructing the financial arrangements of the greater, and we may say of practically the majority, of the voluntary hospitals, throughout the United Kingdom.

Since our last issue Leicester, another county hospital, which throughout the war has shown commendable courage and won for itself the admiration and warm support of the populations which it serves, has had proof of this. It has received a gift of £20,000 from the executors of the late Mr. Thomas G. Langham, whose family has been long and prominently associated with agricultural interests in the county. This gift is free from restrictions and conditions, and the executors suggest that it should be used in the building of an orthopædic ward and an isolation hospital. Now, if agricultural counties are coming to the aid of the hospitals, what may *not* hospitals claim from the cities and towns which have prospered during the war years? Amongst other good things, as we pointed out last week, there is a sum of £285,000 due, if justice is to be done by the Government, to the London hospitals, for money which these hospitals expended in providing for military and naval cases, in addition to the sums already advanced to these institutions during the year 1919. We propose to return to this aspect of the question in a subsequent issue.

Meanwhile we must once more impress upon the managers of all hospitals that it rests with them to show courage, energy, and resource in matters of finance. Every one of them, as we hope, is going carefully into their present position and future requirements. They should then publish the facts, place them before their supporters and the population which they each serve, and pursue a propaganda for help through personal service, increased support, and monetary aid. Such a plan, if pursued with the vigour and energy shown by St. Bartholomew's and other Hospitals, by Leicester, by Cardiff, and by an increasing number of institutions up and down the country, may be relied upon to be attended with equally satisfactory results, to those we have the pleasure to report this week.

We think it well to add, owing to misapprehensions in regard to pensions from the Government, and other matters, which may be in the minds of some who fancy that the taking over of the whole of the voluntary hospitals by the Government would mean advantages of this type to existing holders of important offices, that any such ideas if they exist are chimerical, for the Government has already too many claims to meet to render anything of the kind entertainable. Besides, it is unthinkable that any responsible voluntary hospital official would slacken his or her efforts or interests in the particular institution with which each may be connected, or do less than put forth their utmost efforts to enable it to triumph over the acute financial troubles of the present time. Voluntary hospital troubles can and ought to be faced and successfully overcome. They are already being overcome by some of the best and most exemplary hospitals, as we ventured to forecast, and to-day have the pleasure to demonstrate. "Faint heart ne'er won fair lady," and this has probably never been more true than it is in the period we have now to face and prosper in, providing we show faith and courage enough to win through.

Next let us turn to the explanation of why the life-blood of a number of hospitals is largely dependent on individuals whose responsibility it is to strengthen their faith, and devote themselves entirely to business as faithful stewards of great enterprises. Every voluntary hospital is a great enterprise, and to-day the immediate charge of the financial side of its work never before required, to the same extent, probably, that every man or woman holding such office should, as an absolute necessity, exhibit the highest qualities of resource, enterprise, untiring effort and faithful service. The voluntary hospitals, to be successful and well-administered in these times, must be officered by the very best men and women, whose whole heart and interest is in their work. Committees must realise that to engage an officer at a salary of a few hundred pounds a year and expect him to prove to be a man possessed of the qualities essential to success in financing a great hospital, without the continued co-operation and help of able men and women of influence behind him, who devote themselves continuously to the upbuilding of the resources of their institution, shows a lack of business sense. It exhibits an absence of appreciation of the situation and difficulties of the present time. Every successful hospital must create and carry on an organisation which will prove adequate to provide the monetary aid and supplies essential. Without such supplies few if any hospitals can continue efficient, nor secure the extension and developments necessary to meet the demands of a population, which in the hour of sickness or accident is dependent on them to obtain adequate hospital care, medical treatment, and nursing of the best.

The type of officer which the voluntary hospitals need and must secure in the present day, if they are to continue efficient and prosperous, is a man who is stimulated by the presence of difficulties, and thrives by the call for an output of ever-increasing energy in successfully overcoming them. The days have gone by when anyone who wanted a berth was thought to be at least capable of discharging the duties of a secretary. There are, of course, hospitals where both Committee and staff might be reorganised with advantage. At present the Committees need an intelligent recognition of the circumstances which must, and have to be met and overcome. The financial work entailed upon the secretary of a modern up-to-date hospital may be very heavy. His emoluments should be adequate to the qualities and results which the holder of this office must unfailingly exhibit and achieve. The financing and business affairs of a great hospital are more than adequate to occupy the whole time of a thoroughly trained and competent man. He should be freed from administrative duties outside his department, and so enabled to prove a tower of financial strength to the Committee he may serve. The small finance committee and himself, whilst they retain office, must always prove to be faithful guardians and replenishers of the hospital purse.

The salaries at many hospitals are still inadequate, often dangerously inadequate. But experience demonstrates, that, there is no expenditure which yields a more adequate return in value, than the wise remuneration of the men and women who do the work with unerring efficiency. They secure results which make the institution with which they are connected, a type of all that still takes rank as an exemplar of everything that is best and most efficient, in each and all of its departments. We shall hope that no great hospital in the near future will be handicapped, as at present, by inadequate salaries, and the absence of sufficient strength and enterprise, in any department, to overcome all difficulties that may arise, and especially to meet triumphantly, in proportion to their claims, the needs of its dependent population. We shall welcome information from every hospital which is putting its house in order with the view to overcome the difficulties of the present time. Courage, enterprise, and continuous hard work can secure the essential revenue and equipment, without which neither prosperity, popularity, nor public support will be deserved; nor can they be obtained in the present circumstances.

